# Dose-response effects of resistance training in sarcopenic older adults: systematic review and meta-analysis

**DOI:** 10.1186/s12877-025-06559-4

**Published:** 2025-11-05

**Authors:** Jianxin Ran, Jingfeng Yang, Na Li, Jinqi Yang, Weicheng Yang, Jianxin Chen, Peijie Sun, Yuanpeng Liao

**Affiliations:** 1 Department of Sports Medicine and Health, Chengdu Sport University, Chengdu, 610041 China; 2 Institute of Sports Medicine and Health, Chengdu Sport University, Chengdu, 610041 China; 3 The National Clinical Research Center for Geriatrics, West China Hospital of Sichuan University, Sichuan University, Chengdu, 610041 China; 4 Affliated Hospital of Chengdu Sport University, Chengdu Sport University, No. 2, Tiyuan Road, Wuhou District, Chengdu, Sichuan Province 610041 China

**Keywords:** Exercise, Resistance training, Sarcopenia, Meta-analysis

## Abstract

**Background:**

Currently, resistance training (RT) programs for sarcopenic older adults are numerous they remain controversial, and there is a lack of standardized programs specifically tailored for this population.

**Objectives:**

This study aims to investigate the dose-response effects between RT on grip strength, skeletal muscle mass index (SMI), and short physical performance battery (SPPB) in sarcopenic older adults, to identify key training parameters in RT.

**Methods:**

A comprehensive search was conducted in databases including PubMed, Cochrane Library, Web of Science, China National Knowledge Infrastructure (CNKI), and WANFANG for randomized controlled trials published up to March 2025. Studies were selected based on the inclusion and exclusion criteria outlined in this study, resulting in 12 studies. The Grades of Recommendation, Assessment, Development, and Evaluation (GRADE) system was used to assess the quality of evidence, while Cochrane’s Risk of Bias Tool evaluated the methodological quality of the included studies. Meta-analysis of baseline data and outcomes was performed using Review Manager version 5.3.4 and R version 3.5 software.

**Results:**

12 studies involving 538 participants met the inclusion criteria. Compared to the control group, RT had a moderate effect on grip strength (SMD = 0.63, 95% CI 0.43–0.83; I² = 32%, X² = 11.73, df = 8, *p* < 0.00001) and SPPB (SMD = 0.56, 95% CI 0.18–0.94; I² = 53%, X² = 8.48, df = 4, *p* = 0.004) with in sarcopenic older adults, but it did not significantly improve SMI (SMD = 0.24, 95% CI -0.05-0.53; I² = 0%, X² = 2.85, df = 4, *p* = 0.10). Meta-regression analysis revealed that training frequency (*p* < 0.0001) and training intensity (*p* = 0.0019) were significant predictors of grip strength improvement, while training frequency (*p* = 0.052) may be a potential predictor for SPPB improvement. Additionally, subgroup analysis showed that incorporating pulling exercises into the training program significantly improved grip strength compared to studies that did not include such exercises (SMD = 0.90, 95% CI 0.61–1.19; I² = 0%, X² = 0.75, df = 2, *p* < 0.00001).

**Conclusions:**

Our meta-analysis suggests that RT is associated with improvements in grip strength and SPPB scores in sarcopenic older adults, whereas its effect on SMI was not statistically significant. The findings indicate that performing moderate-intensity resistance training twice a week is a promising strategy for improving grip strength, while a similar frequency may also benefit SPPB scores. Furthermore, incorporating pulling exercises into the RT regimen may positively influence grip strength outcomes.

**Supplementary Information:**

The online version contains supplementary material available at 10.1186/s12877-025-06559-4.

## Introduction

Sarcopenia is a syndrome characterized by the progressive loss of skeletal muscle mass, strength, and physical function due to age-related changes [1, 2]. It is highly prevalent among older adults, with prevalence rates of approximately 9% to 10% in community-dwelling individuals, 30% to 50% in institutionalized patients, and 23% to 24% in hospitalized patients [3, 4]. Sarcopenia is associated with a significantly increased risk of adverse outcomes, such as dysphagia, cognitive impairment, fractures, falls, hospitalization, and all-cause mortality. These risks are notably amplified in the geriatric population [4, 5]. Therefore, preventing and treating sarcopenia is crucial for enhancing physical function and health in older adults.

Currently, no specific pharmacological interventions have been widely recognized for treating sarcopenia. Some medications, such as selective androgen receptor modulators (SARMs), estrogen, and dehydroepiandrosterone (DHEA), are used in sarcopenia management, but their effects require further validation [6, 7]. Sarcopenia is a multifactorial condition, and focusing solely on increasing muscle mass or strength may not be sufficient to improve overall physical function [7].

Exercise is recognized as the preferred nonpharmacological strategy for managing sarcopenia [8]. Physical activity mitigates age-related oxidative stress and chronic inflammation and potentially enhances mitochondrial function and insulin sensitivity [9]. Resistance training (RT) promotes muscle hypertrophy by stimulating the AKT-mTOR protein signaling cascade, which improve protein synthesis and reduces protein catabolism [10]. RT has also been shown to increase physical function parameters, including walking speed, grip strength, and balance [11].

However, standardized programs for RT specifically tailored to sarcopenic older adults are lacking [12]. Current studies often involve multi-component exercises or combined training, or RT with nutritional support, which may affect the accuracy of RT outcomes. Additionally, sarcopenic older adults often have comorbid severe chronic diseases that may confound dose-response results. There is a lack of studies on the dose-response relationship of pure RT in sarcopenic older adults without other chronic diseases. Therefore, this study aims to explore the dose-response relationship between RT and grip strength, skeletal muscle mass index (SMI), and short physical performance battery (SPPB) through meta-regression, providing evidence for more targeted training programs for older adults with sarcopenia.

## Methods

This meta-analysis is registered with the International Prospective Register of Systematic Reviews (PROSPERO: 42023463685). This meta-analysis adheres to the methodological standards recommended by the Cochrane Collaboration and conforms to the ‘Preferred Reporting Items for Systematic Reviews and Meta-Analyses (PRISMA) guidelines [13].

### Eligibility criteria

By utilizing the population, intervention, comparison, outcome, and study design (PICOS) framework [14], the studies that met the following criteria were included: (a) population: sarcopenic older adults, aged 60 years or older defined according to the European Working Group on Sarcopenia in Older People (EWGSOP) and the Asian Working Group for Sarcopenia (AWGS), or similar criteria, as well as individuals with sarcopenic obesity; (b) intervention: the intervention group received any type of RT; (c) comparator: control groups engaged in standard care without physical activity interventions (e.g., health education); (d) outcome: at least one measure of physical function [e.g., short physical performance battery (SPPB)] and muscle mass [e.g., skeletal muscle mass index (SMI) and muscle strength (e.g., grip strength); (e) study design: randomized controlled trials (RCTs); (f) evaluation of RT intensity, assessed via the repetition maximum (RM) scale or the Borg Rating of Perceived Exertion (RPE) scale. (g) The included literature can be in either English or Chinese. We excluded studies that met the following criteria: (a) inadequate reporting of results (mean and standard deviation); (b) they included sarcopenic older adults and reporting comorbid chronic conditions (e.g., hypertension, diabetes, heart disease); (c) incorporated multicomponent exercise interventions (e.g., combined RT and endurance training); (d) involved nutritional supplementation (e.g., protein, creatine).

### Information sources and search strategy

This study conducted a systematic review by searching core journals indexed in databases such as PubMed, Cochrane Library, Web of Science, CNKI, and WANFANG for randomized controlled trials published up to March 2025. Studies in both English and Chinese languages were considered eligible. Search strategies in the databases were formulated by utilizing relevant and validated keywords, including MeSH terms, and combining them using OR and AND operators. The search strategy, exemplified by the PubMed database, includes several key components:

(1) Exercise Therapy Keywords: A comprehensive set of exercise-related keywords was employed, encompassing terms such as “exercise therapy,” “exercising,” “physical activity,” “acute exercise,” “isometric exercise,” “aerobic exercise,” and “exercise training,” along with their synonyms and associated phrases. This approach ensures a thorough coverage of the various facets of exercise therapy. (2) Sarcopenia: Studies related to sarcopenia were identified by searching for “sarcopenia” or “sarcopenias” in the titles or abstracts. (3) Randomized Controlled Trials: The search was refined to include only randomized controlled trials by using “randomized controlled trials as topic” and relevant MeSH terms, as well as keywords like “random.” Additionally, terms for single-blind and double-blind methodologies were incorporated to ensure the scientific validity and reliability of the trials identified. This methodical search strategy is designed to systematically retrieve high-quality studies that investigate the role of exercise therapy in sarcopenia. (For complete search strategies, refer to Supplement 1.)

### Selection process and data collection process

Two independent reviewers, (F C and Y Z), assessed the titles and abstracts of all identified studies. Any initial disagreements were resolved through discussion and consensus to ensure strict adherence to the predefined inclusion and exclusion criteria. If consensus was unattainable, a third reviewer (JX. C)was consulted for an impartial decision, which was considered final. This method maintained the systematic review’s rigor, with all decisions meticulously documented. Extracted data underwent cross-verification for consistency and completeness. Inconsistencies were resolved by contacting study authors for additional verification or clarifying published information. Post-discrepancy resolution, results were synthesized and analysed per the review’s predefined objectives. Extracted variables included group demographics, age, intervention details, training parameters, and outcomes. Corresponding authors of included studies were contacted for supplementary information when necessary. Studies were excluded if authors didn’t respond or required information was unavailable. The literature review followed the PRISMA flowchart, recording the number of included studies at each phase and detailing exclusion rationales.

Specifically, F C and Y Z worked independently to evaluate the studies, with their assessments compared for consistency. When discrepancies arose, they engaged in discussions to reach a consensus. In rare cases where consensus couldn’t be reached, J X C provided a decisive and impartial ruling. For data extraction, F C and Y Z cross-checked the extracted information against the original studies. When inconsistencies were identified, they first attempted to resolve them by rechecking the source material. If this didn’t suffice, they directly contacted the corresponding authors of the studies for clarification or additional details. All communications and decisions were thoroughly documented throughout the process. When contacting authors, clear and concise requests were made, specifying exactly what information was needed and why. If authors failed to respond within a reasonable timeframe or couldn’t provide the necessary data, the study in question was excluded from the final analysis. This approach ensured that all decisions regarding study inclusion, data extraction, and handling of missing data were transparent, well-documented, and aligned with the review’s predefined protocol, thereby enhancing the overall transparency and scientific integrity of the systematic review.

### Study risk of bias assessment

Any discrepancies between the two reviewers (YF. L and JL. Z) were resolved through discussion and consultation with a third reviewer (JF. Y), who provided an impartial evaluation to ensure consistency and objectivity. If the third reviewer’s decision was still disputed, a final consensus meeting was held to reach an agreement. This process maintained the integrity of the bias risk assessment and ensured that all classifications were made transparently and consistently. Each study was assessed for the risk of bias in the following domains: selection bias, performance bias, detection bias, attrition bias, and reporting bias. The results of the risk of bias evaluation were used to categorize each study as either low risk, unclear risk, or high risk of bias [15]. This classification provided valuable insight into the overall quality and reliability of the evidence included in the systematic review. The Review Manager version 5.3.4 software (Copenhagen: Nordic Cochrane Centre, Cochrane Collaboration, 2008) facilitated this process, allowing the reviewers to systematically evaluate and document the risk of bias for each study. This rigorous approach ensured that the potential for bias was considered at every stage of the review, contributing to the reliability and validity of the final synthesis.

### Certainty of evidence

The quality of evidence was appraised with the Grades of Recommendation, Assessment, Development, and Evaluation (GRADE) system, facilitating the categorization of evidence quality pertaining to outcomes and absolute risk reduction [16]. The GRADE system accounts for five criteria that may downgrade the evidence quality and three criteria that may upgrade it, assessing the quality of evidence with consideration of the risk of bias, inconsistency, indirectness, imprecision, and publication bias. The evidence quality is thus rated as high, moderate, low, or very low.

### Exercise intensity grading

In accordance with the guidelines established by the American College of Sports Medicine, the intensity of RT was classified into five distinct categories: very light, light, moderate, vigorous, and near-maximal to maximal intensity. These categories were determined based on either RM or RPE [17]. RM refers to the heaviest weight that can be lifted for a complete single rep of a given exercise [18]. The RPE, which is subjective, serves as a reliable indicator of RT intensity [19]. However, the sarcopenia management guidelines advocate for RT of at least moderate intensity [20].Accordingly, we stratified RT intensity into three distinct levels: light to moderate (LMRT), moderate (MRT), and moderate to vigorous (MVRT). For LMRT, Borg RPE scale scores range from 6 to 11 or are less than 49% of the 1RM; MRT corresponds to Borg RPE scores of 12–13; MVRT is defined by scores of 14–17 on the Borg RPE scale, which corresponds to 70%−84% of the 1RM [21].

### Synthesis methods

To ascertain the effects and dose‒response relationships of RT in sarcopenic older adults, calculations of standardized mean differences (SMD) and their corresponding 95% confidence intervals (CIs) were performed to evaluate the influence of RT on physical function. The corresponding authors were contacted to obtain data when the extraction of mean values and standard deviations (SDs) was not feasible. Studies for which author contact was unsuccessful were excluded from the analysis. Using Review Manager version 5.3.4 software, SMD such as grip strength and SMI, were computed to assess the reported outcomes of the interventions. A positive SMD value indicates a beneficial effect of RT compared with the control group. The meta-analysis was conducted with Review Manager version 5.3.4 software; random effects models and fixed effects model were used to determine the efficacy of RT interventions, if I² ≥50%, the random effects model should be used; if I² < 50%, the fixed effects model should be used. Forest plots were constructed to depict the pooled effects and the corresponding standardized mean differences with 95% CI. Per Cohen’s benchmarks, SMD values ranging from 0.00 to 0.49 denote a small effect, those from 0.50 to 0.79 signify a moderate effect, and values of 0.80 or above represent a large effect [22]. Heterogeneity was quantified via the I² statistic and the X² test.

For the sensitivity analysis, we first examined the differences in effect sizes of the outcome measures using both fixed-effects and random-effects models. Then, we employed the leave-one-out method to identify the sources of heterogeneity among the studies with differing effect sizes. Publication bias was assessed by examining the symmetry of the funnel plot. Given the diversity of RT programs in the studies, and the potential for different exercise arrangements to exert varying effects on grip strength. We conducted the first subgroup analyses aimed to explore the impact of incorporating pulling exercises (e.g., lat pulldown, deadlift, and seated row) into the intervention plan compared to interventions that did not include pulling exercises on grip strength. Given that sarcopenic obese individuals were included in the study population, the second subgroup analysis aimed to investigate the effects of RT on both sarcopenic obese patients and those with sarcopenic older adults. Since grip strength is one of the most critical indicators for assessing sarcopenia, we conducted subgroup and sensitivity analyses on studies that included grip strength as an outcome measure. Additionally, sensitivity analyses were performed on studies that included SMI and SPPB as outcome measures. These analyses were conducted to further evaluate the impact of RT on sarcopenic obese patients and those with sarcopenic older adults.

Meta-regression analysis was conducted using R version 3.5 (The R Foundation for Statistical Computing, Vienna, Austria) to identify potential moderators of the RT effect, including training period (in weeks), training frequency (number of training sessions per week), volume per exercise (calculated as the maximum number of sets multiplied by the maximum number of repetitions per session), and intensity (LMRT, MRT, and MVRT). A *p*-value of < 0.05 was considered indicative of statistical significance for all analyses.

## Results

### Study selection

**Fig. 1:**
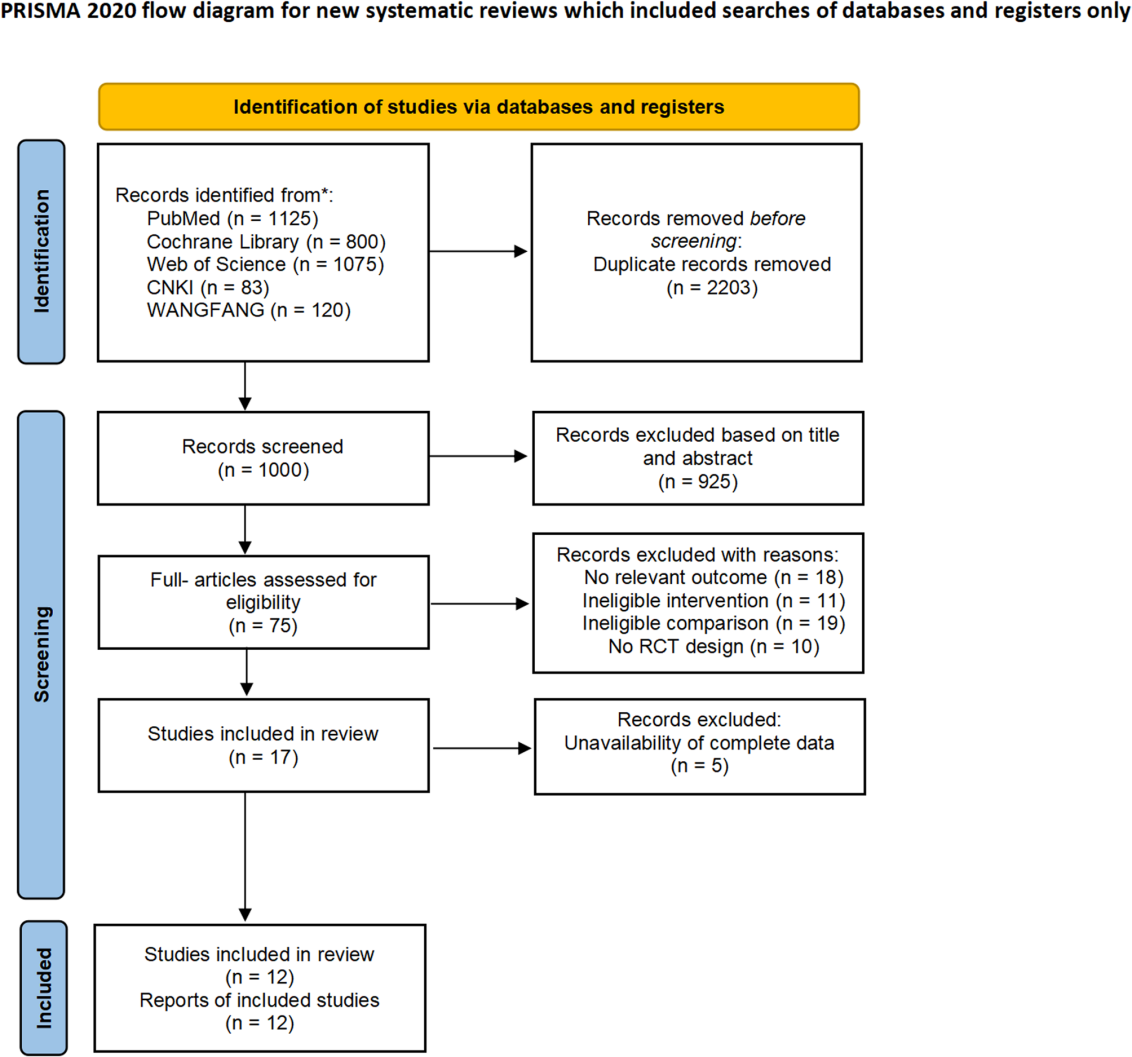
The flowchart for the study screening and selection process according to the PRISMA guidelines

After the removal of duplicate records and application of the inclusion criteria, a total of 1000 studies were identified from the electronic database searches (Fig. 1). After the screening of citations by title and abstract, 75 potentially eligible studies were chosen for full-text review. In the final stage of selection, 12 studies were included in the analysis.

### Study characteristics

Table 1 presented a summary of the characteristics of the RT across the included studies. In these studies, two studies involved participants from Asian populations [23, 24], while the remaining studies involved participants from Western populations. A total of 538 participants were included across the studies. With respect to participant gender, six studies exclusively featured female participants [23–28], and two studies did not report subject gender [29, 30]. The interventions included the use of elastic bands in two studies [23, 24], a weighted vest in one study [27], kettlebell machines in one study [25], and weight machines in the remaining nine studies [26–34]. The frequency of RT was between two and three sessions per week. The training period for each study was no less than eight weeks and no longer than 26 weeks. Repetitions in RT were predominantly set at approximately 10 (ranging from 3 to 20 repetitions), the maximum number of sets was 8, and the minimum was between 2 and 3 sets.

**Table 1:** Characteristics of the RT of the studies included in the quantitative synthesis

study	Age(years)	N	sex(men/women)	training intensity	Resistancemode	Training frequency (weeks)	Training period(weeks)	Rest timeBetween sets(seconds)	Repetitionsper set	Sets per exercise
Otsuka 2022 [31]	RT:63.6 ± 8.1 CG:63.5 ± 8.5	RT:33 CG:17	RT:16/17 CG:9/8	MRT（60%1RM）	weightmachines	3/week	24	120 s	14reps	3
Chen 2018 [25]	RT: 66.7±5.3 CG: 68.3±2.8	RT:17 CG:16	Only women	MRT（60-70%1RM）	kettlebell	2/week	8		8-12reps	3
Hassan 2016 [30]	RT: 85.7±7.0 CG:70.0 ± 0.29	RT:20 CG:21	unknown	MRT（Borg 12-14）	weightmachines	2/week	8		8-12reps	2-3
Liao 2017 [23]	RT: 66.39±4.49 CG: 68.42±5.86	RT:25 CG:21	Only women	MRT（Borg 13）	elastic bands	3/week	12		10reps	3
Vasconcelos 2016 [26]	RT: 72±4.6 CG: 72±3.6	RT:14 CG:14	Only women	MRT（50-75%1RM）	weightmachines	2-3/week	10	30-60 s	8-12reps	2-3
Tsekoura 2018 [32]	RT: 74.56±6.04 CG: 72.89±8.31	RT:18 CG:18	RT:2/16 CG:2/16	MRT（Borg 12）	Weightmachines	3/week	12		8-12reps	2-3
Zech 2012 [33]	RT: 77.4±6.2 CG: 75.9±7.8	RT:18 CG:18	RT:2/16 CG:2/16	LRT（Borg 10-12）	weightmachines	2/week	12	120 s	15reps	2
Hamaguchi 2017 [27]	RT: 60.4±2.7 CG: 60.6±2.3	RT: 7 CG: 8	Only women	MRT (Borg 13)	weighted vest	3	6	15	3	8
Liao 2018 [24]	RT: 68.32±6.05 CG: 66.67±4.54	RT: 33 CG: 23	Only women	MRT (Borg 13)	elastic bands	3	12		10-20	3
Rufino 2023 [28]	RT: 79.9±7.2 CG: 79.6±7.7	RT: 20 CG: 18	Only women	MVRT (70%1RM)	weight machines	2	26		10-15	3
Sahin 2018 [29]	RT: 84.18±6.85 CG: 85.37±4.7	RT: 16 CG: 16	unknown	MVRT (70%1RM)	weight machines	3	8		6-10	
Tieland 2015 [34]	RT: 78.4±1.0 CG: 79.5±1.0	RT: 62 CG: 65	RT: 21/41 CG: 30/35	MRT (50-75%1RM)	weight machines	2	24	60	10-15	3

*RT *Resistance Trainig, *CG* Control Group, *LRT* Low-Resistance Training, *MRT* Moderate Resistance Training, *MVRT* Moderate to Vigorous Training Resistance

### Risk of bias in studies

**Fig. 2:**
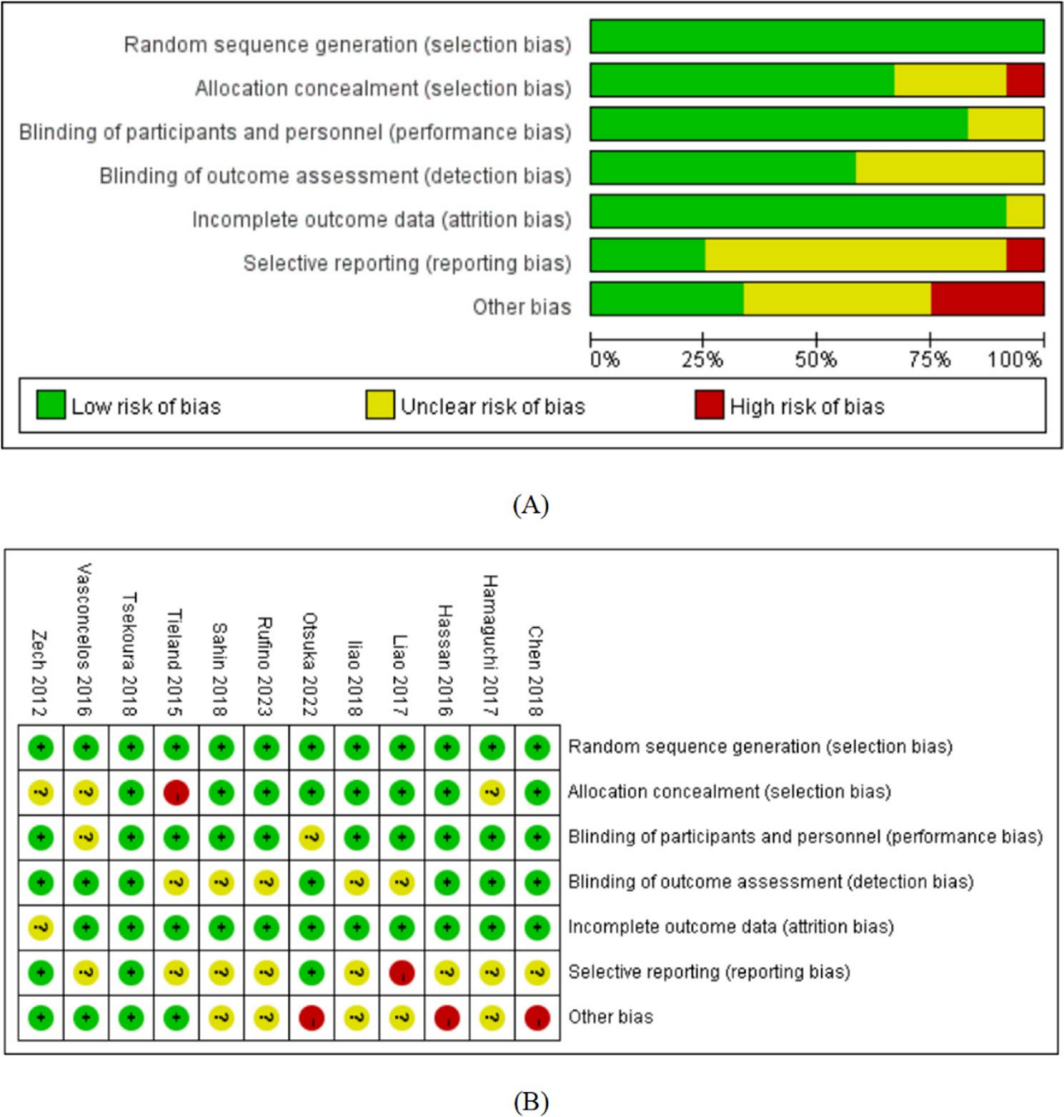
**A** Percent of studies with categories for risk of bias. **B** Summary of the risk of bias in each study

The detailed risk of bias assessment for the included studies was depicted in Fig. 2. For the domain of random sequence generation, selection bias was low in 12 studies. With respect to allocation concealment, the risk of bias was unclear in three studies [26, 27, 33], high in one study [32], and low in eight studies [23–25, 28–32]; for the blinding of participants and personnel, the performance bias was unclear in two studies [26, 31], high in three studies [25, 29, 32], and low in seven studies [23, 24, 27, 28, 30, 33, 34]; for the blinding of outcome assessment, the detection bias was unclear in five studies [23, 24, 28, 29, 34] and low in seven studies [25–27, 30–33]; for incomplete outcome data, the attrition bias was unclear in one study [33] and low in 11 studies [23–32, 34]; for selective reporting, the reporting bias was unclear in eight studies [24–30, 34], low in three studies [31–33], and high in one study [23]; and for other bias, the risk of bias was low in four studies [26, 32–34], high in three studies [25, 30, 31], and unclear in five studies [23, 24, 27–29]. The included studies predominantly presented an unclear risk of bias with respect to selective reporting, participant blinding, and other potential biases. They presented a low risk of bias in aspects such as random sequence generation, handling of incomplete outcome data, and reporting.

### Results of syntheses-effectiveness of RT

#### Effects of RT on grip strength

**Fig. 3:**
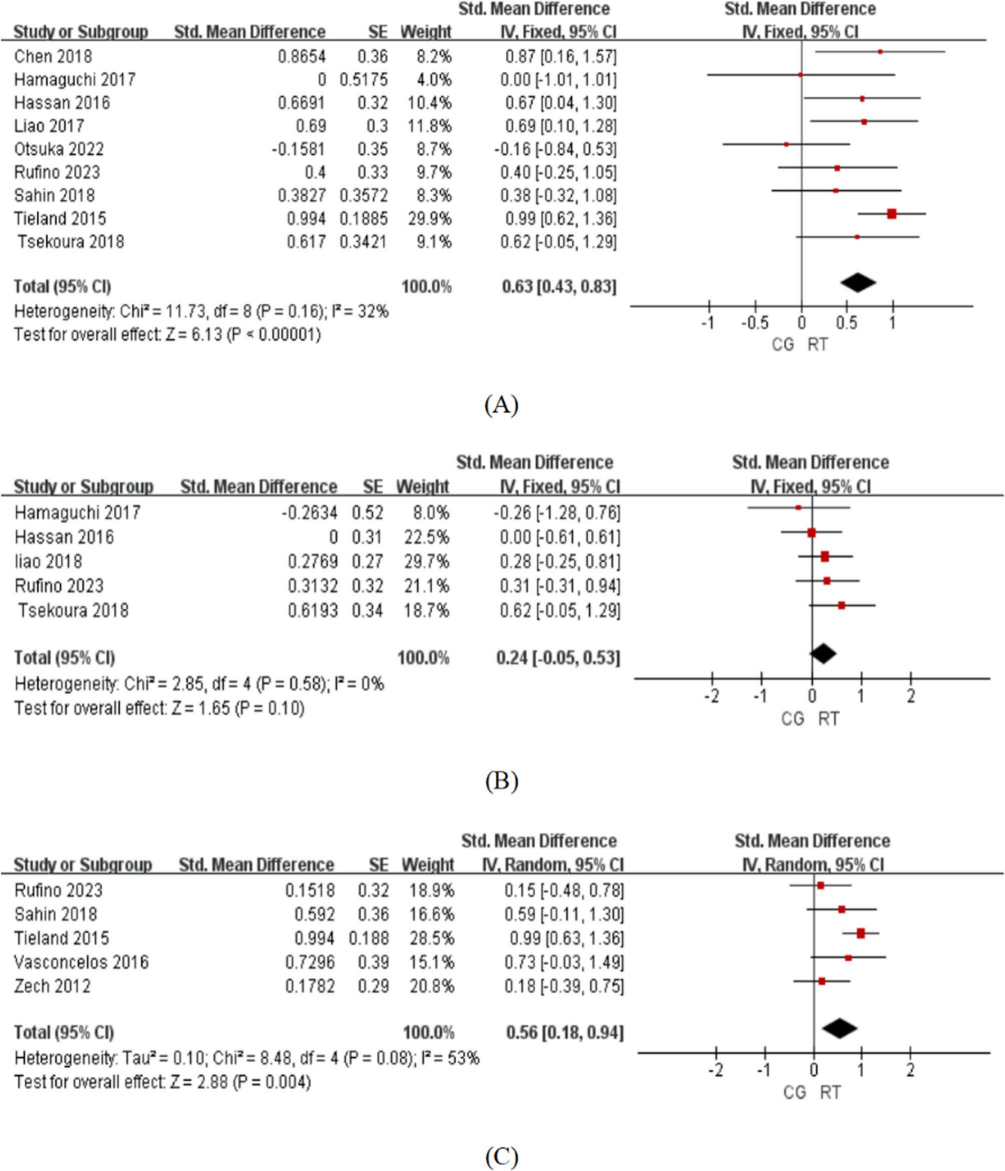
**A** Grip strength. **B** SMI: skeletal muscle mass index. **C** SPPB: short physical performance battery. CG control group, CI confidence interval, IV inverse variance, RT resistance training, SE standard error, SMD standardized mean difference

All nine studies demonstrated positive effects of RT on grip strength, with SMD of 0.63 (95% CI 0.43–0.83; I² = 32%, X² = 11.73, df = 8, *p* < 0.00001), indicating that RT has a medium effect on grip strength. The results of the meta-analysis using the fixed effects model showed a statistically significant overall difference between the RT group and the control group, with low heterogeneity. (Fig. 3).

The first subgroup analyses were conducted to compare studies that included resistance training exercises, such as lat pulldowns, deadlifts, and seated rows [23, 25, 34]. The results indicated that the group using pull-type exercises with a SMD of 0.90 (95% CI 0.61–1.19; I² = 0%, X² = 0.75, df = 2, *p* < 0.00001) performed significantly better than the group not using pull-type exercises with a SMD of 0.38 (95% CI 0.10–0.66; I² = 0%, X² = 4.45, df = 5, *p* = 0.009).

Another subgroup analysis showed that RT can improve the grip strength of sarcopenic obese older adults with a SMD of 0.76 (95% CI 0.31–1.21; I² = 0%, X² = 0.14, df = 1, *p* < 0.001) and sarcopenic older adults with a SMD of 0.50 (95% CI 0.17–0.82; I² = 46%, X² = 11.19, df = 6, *p* = 0.003).

#### The results of the meta-analysis using the fixed effects model showed no statistically significant difference in SMI between the RT group and the control group

The heterogeneity was low. (SMD = 0.24, 95% CI −0.05-0.53; I² = 0%, X² = 2.85, df = 4, *p* = 0.10).

#### Impact of RT on SPPB

A medium effect of RT on SPPB scores was observed, the results of the meta-analysis using the random effects model showed a statistically significant overall difference between the RT group and the control group, with moderate heterogeneity. (SMD = 0.56, 95% CI 0.18–0.94; I² = 53%, X² = 8.48, df = 4, *p* = 0.004).

### Dose‒response relationships

Meta-regression analysis indicates that intensity (*p* = 0.0019) and training frequency (*p* < 0.0001) are intervention factors influencing the effect of RT on grip strength. training frequency (*p* = 0.052) maybe a potential predictor of RT’s effect on improving SPPB. Specifically, training twice a week has a significant positive effect on grip strength; while training three times a week may not significantly improve grip strength and could even have a slight negative impact. Moderate-intensity RT may be sufficient to produce significant effects, whereas the effect of moderate-to-vigorous intensity on grip strength appears to be limited. Training twice a week has the effect on SPPB that is statistically marginal, suggesting a potential influence. Compared to training twice a week, training three times a week does not have a significant additional effect on the SPPB. Additionally, training frequency, training period, exercise volume per session, and training intensity are not associated with improvements in SMI. (Table 2)

**Table 2:** Meta-regression of moderators of the effects of resistance training on grip strength, SMI and SPPB

Outcome	Number of comparisons	Estimate	95% CI	*P* value
Grip strength				
Training period	9	0.0018	(-0.0343-0.0379)	0.92
Training frequency	9	0.7979	(0.4968-1.0990)	<0.01
Volume per exercise	9	0.0082	(-0.0249-0.0413)	0.63
Intensity	5	0.4748	0.1746-0.7749	<0.01
SPPB				
Training period	5	-0.0051	(-0.0450-0.0348)	0.80
Training frequency	5	0.5016	(-0.0056-1.0089)	0.05
Volume per exercise	5	0.0181	(-0.0377-0.0739)	0.52
Intensity	5	0.4897	(-0.3935-1.3730)	0.28
SMI				
Training frequency	5	0.1665	(-0.4202-0.7532)	0.58
Training period	5	-0.0051	(-0.0450-0.0348)	0.80

SMD: Standardized Mean Difference; SMI: skeletal muscle mass index; SPPB: short physical performance battery

### Sensitivity analysis

We investigated the effect sizes under both fixed-effects and random-effects models and found minimal differences (Table 3) Subsequently, sensitivity analysis was conducted by sequentially excluding individual studies from the nine studies included in the grip strength and the five studies included in the SPPB. It was found that one study contributed to increased heterogeneity [34]. After excluding this study, the heterogeneity of grip strength decreased from (SMD = 0.63, I² = 32%) to (SMD = 0.48, I² = 0%), while the heterogeneity of studies like SPPB decreased from (SMD = 0.56, I² = 53%) to (SMD = 0.36, I² = 0%).This study had a relatively large sample size and a higher proportion of male participants in both the RT and control groups, which likely contributed to the observed heterogeneity (Table 4).

**Table 3:** Sensitivity analyses of the included studies

Outcome	Models	Relative effect	95% CI	*P*-value	I^2^
Grip strength	Random effect	0.63	[0.43, 0.83]	< 0.001	32%
	fixed effect	0.58	[0.32, 0.83]	< 0.001	32%
SMI	Random effect	0.24	[-0.05, 0.53]	0.10	0%
	fixed effect	0.24	[-0.05, 0.53]	0.10	0%
SPPB	Random effect	0.56	[0.18, 0.94]	< 0.01	53%
	fixed effect	0.64	[0.40, 0.88]	< 0.001	53%

*SMD* Standardized Mean Difference, *SMI* Skeletal muscle mass index, *SPPB* Short physical performance battery

**Table 4:** Summary table of sensitivity analysis

Excluded Article	SMD AfterRemoval	95% CI of Effect SizeAfter Removal	I² AfterRemoval (%)	OriginalSMD	Original95% CI of Effect Size	OriginalI² (%)
Grip strength						
Chen 2018	0.54	(0.26,0.82)	38%	0.58	(0.32,0.83)	32%
Hamaguchi 2017	0.61	(0.35,0.87)	31%	0.58	(0.32,0.83)	32%
Hassan 2016	0.55	(0.26,0.84)	40%	0.58	(0.32,0.83)	32%
Liao 2017	0.55	(0.25,0.84)	40%	0.58	(0.32,0.83)	32%
Otsuka 2022	0.71	(0.50,0.92)	0%	0.58	(0.32,0.83)	32%
Rufino 2023	0.59	(0.31,0.87)	37%	0.58	(0.32,0.83)	32%
Sahin 2018	0.59	(0.31,0.87)	37%	0.58	(0.32,0.83)	32%
Tieland 2015	0.48	(0.24,0.72)	0%	0.58	(0.32,0.83)	32%
Tsekoura 2018	0.56	(0.27,0.85)	40%	0.58	(0.32,0.83)	32%
SPPB						
Rufino 2023	0.66	(0.26,1.06)	48%	0.56	(0.18,0.94)	53%
Sahin 2018	0.54	(0.07,1.01)	65%	0.56	(0.18,0.94)	53%
Tieland 2015	0.36	(0.03,0.69)	0%	0.56	(0.18,0.94)	53%
Vasconcelos 2016	0.52	(0.05,0.98)	64%	0.56	(0.18,0.94)	53%
Zech 2012	0.67	(0.27,1.06)	44%	0.56	(0.18,0.94)	53%

*SMD* Standardized Mean Difference, *SPPB* short physical performance battery, *CI* Confidence interval

### Publication bias test

**Fig. 4:**
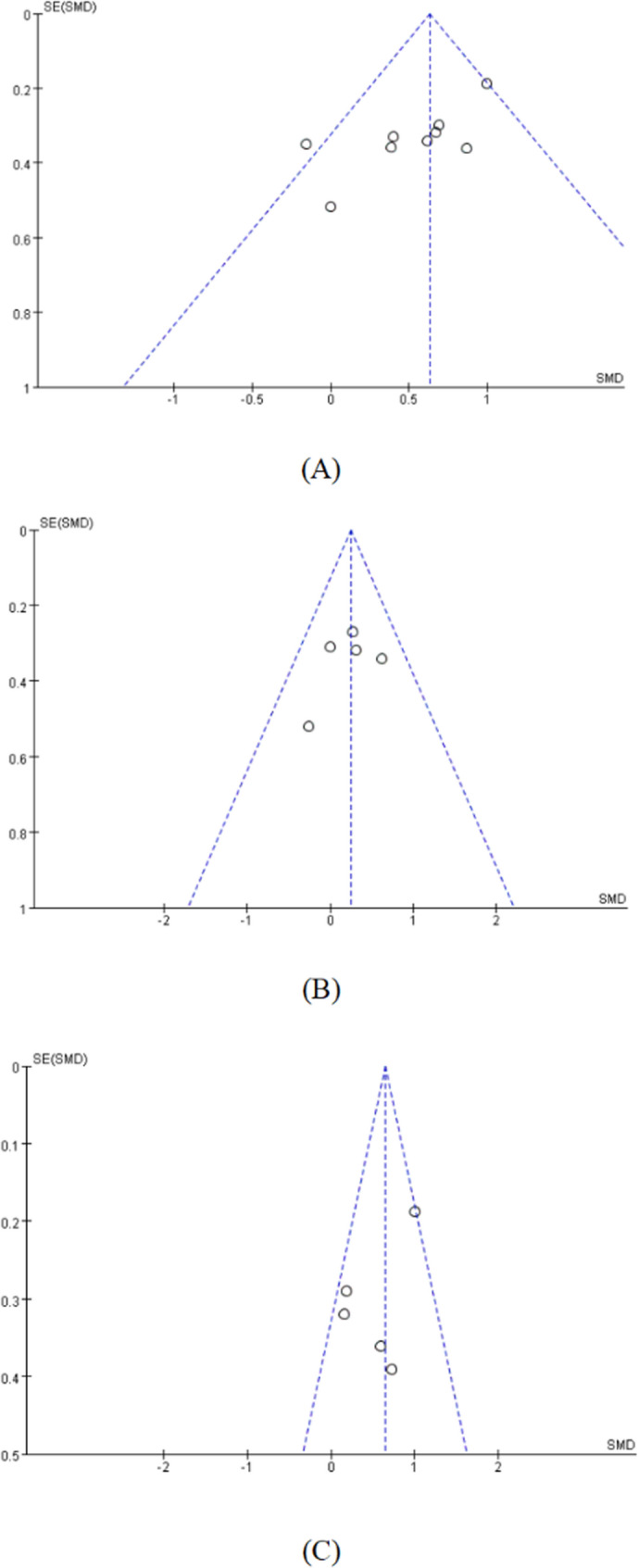
Funnel Plot (**A**) Grip strength. **B** SMI: skeletal muscle mass index. **C** SPPB: short physical performance battery. SE: Standard Error. SMD: Standardized Mean Difference

The funnel plot of the meta-analysis assessing the effects of RT on grip strength, SMI, and SPPB in sarcopenic older adults showed an overall asymmetrical distribution of data points, although some were symmetrically distributed. To further assess the potential publication bias in the studies, an Egger test was conducted. The p-values for grip strength, SMI, and SPPB were 0.0118, 0.4903, and 0.0602, respectively However, after excluding studies that incorporated pulling exercises in their training program [23, 25, 34], the publication bias became non-significant (*p* = 0.3752), suggesting that the use of pulling exercises in RT programs may be a major contributor to the observed publication bias.(Fig. 4).

### Certainty of evidence

Based on the GRADE assessment, the overall quality of evidence was deemed low. The certainty of the evidence for the evaluated outcomes, namely, grip strength, SMI, and SPPB, is classified as low. Regarding the risk of bias, a significant risk was noted for the outcomes of grip strength, SMI, and SPPB. All studies were evaluated as low risk in terms of inconsistency, indirectness, and imprecision (for detailed information, see Table 5).

**Table 5:** GRADE profile of the included studies

Dose-Response Relationships of Resistance Training in older adults with sarcopenia: A Systematic Review and Meta-Analysis.						
Patient or population: patients with sarcopenia Settings: Dose-Response Relationships of Resistance Training in older adults with sarcopenia Intervention: Resistance Training Comparison: health education						

Outcomes	Illustrative comparative risks* (95% CI)		Relative effect (95% CI)	No of Participants (studies)	Quality of the evidence (GRADE)	Comments
	Assumed risk control	Corresponding risk Resistance Training				
grip strength		The mean grip strength in the intervention groups was 0.63 standard deviations higher (0.43 to 0.83 higher)		418 (9 studies)	⊕⊕⊝⊝ low	
SMI		The mean smi in the intervention groups was 0.24 higher (0.05 to 0.53 higher)		186 (5 studies)	⊕⊕⊝⊝ low	
SPPB		The mean sppb in the intervention groups was 0.56 standard deviations higher (0.18 to 0.94 higher)		261 (5 studies)	⊕⊕⊝⊝ low	

*CI* Confidence interval

^*^The basis for the assumed risk (e.g. the median control group risk across studies) is provided in footnotes. The corresponding risk (and its 95% confidence interval) is based on the assumed risk in the comparison group and the relative effect of the intervention (and its 95% CI)

## Discussion

This meta-analysis evaluated the overall effects of RT on grip strength, SMI, and SPPB. The results from 12 eligible randomized controlled trials indicated that RT had a moderate effect on grip strength and SPPB, while RT did not improve the SMI in sarcopenic older adults. We performed a meta-regression analysis to assess the impact of training variables (e.g., training period, frequency, volume per exercise, and intensity). on the effectiveness of RT. The results showed that frequency (*p* < 0.0001) and training intensity (*p* = 0.0019) were significant predictors of improvement in grip strength, while frequency (*p* = 0.052) may be an important influencing factor for improvement in SPPB.

### Grip-strength

Subgroup analysis revealed that incorporating pulling movements into the RT program significantly improved grip strength [23, 25, 34], This benefit appears to be driven by the isometric phase inherent in pulling exercises: grasping a barbell forces the forearm flexors and intrinsic hand muscles to sustain a prolonged isometric contraction, providing a potent stimulus for grip-strength gains. Programmes without pulling movements did not elicit comparable improvements. Cima et al. [35]. likewise reported that targeted grip training enhances performance. Because only a small number of studies contributed to this comparison, the statistical power is limited; the finding should therefore be interpreted cautiously.

The second subgroup analysis indicated that RT also benefits sarcopenic obese older adults, improving both grip strength and functional measures such as the Timed Up and Go [36, 37]. Consistent with previous work, the inclusion of sarcopenic obese individuals did not attenuate RT-induced gains in grip strength, suggesting that obesity status does not meaningfully modify this response in sarcopenic older adults. This finding is further supported and expanded upon by a recent systematic review and meta-analysis by Gonçalves et al. which specifically investigated exercise training in older adults with sarcopenic obesity [38]. Their findings corroborate that exercise training, including RT, is effective in this population, demonstrating significant improvements not only in muscle strength (both upper and lower limb) and physical performance (e.g., chair stand test) but also in reducing body fat percentage. Notably, resistance training was specifically highlighted as an effective modality for reducing fat mass and improving strength outcomes [38]. This comprehensive analysis strengthens the conclusion that RT confers broad benefits for older adults with sarcopenic obesity, targeting key components of the condition including fat mass, muscle strength, and functional capacity.

### Skeletal muscle mass index

Compared to grip strength and SPPB tests, SMI reflects changes in body composition rather than functional capacity, and is therefore more likely to respond when RT is paired with nutritional support. In the study by Osuka et al. [39], sarcopenic participants performed RT twice weekly and consumed milk daily; after 12 weeks, SMI had increased significantly. Similarly, a trial that provided whey protein (1.2–1.5 g·kg⁻¹·day⁻¹) without RT still produced a meaningful rise in appendicular muscle-mass index (AMMI) after 12 weeks [40]. Our review was restricted to studies of RT alone, which may explain the absence of an SMI benefit.

A robust literature shows that RT combined with nutrition enhances lean mass in older adults. Supplementing 25–30 g of protein per meal for 24 weeks increased muscle mass relative to placebo [41]. Additionally, the combination of and adding creatine to RT exerts a synergistic effect in sarcopenic elders [42]. Comparable benefits have been reported with leucine-enriched whey plus RT [43]. Adequate protein ingestion during RT heightens the muscle’s anabolic sensitivity, improving net protein balance and, ultimately, lean-tissue accretion [44]. Thus, RT plus targeted nutrition appears to be the most effective strategy for raising SMI in sarcopenic individuals.

### Short physical performance battery

The results of the study showed that RT produced a moderate overall improvement in SPPB scores (SMD = 0.56; 95% CI = 0.34–0.78), However, substantial heterogeneity was observed (I² = 53%). When the study by Tieland et al. [34] was excluded, heterogeneity was eliminated (I² = 0%) and the pooled effect size decreased to SMD = 0.36 (95% CI = 0.18–0.54). The Tieland study is unique among the included trials: it enrolled 127 participants and maintained an almost equal male-to-female ratio, whereas two other studies recruited only women [24, 28], and another included only four men [33]. Walking speed—the SPPB component most responsive to RT—improves more in men than in women [45]. Consequently, the balanced sex distribution and larger sample size in Tieland et al. inflated the overall effect size but simultaneously introduced heterogeneity.

### Dose-response

Our results diverge from those of Cristiane et al., who concluded that three sessions per week maximised SPPB gains. In our cohort, two sessions produced the larger effect (*p* = 0.0526). Optimal frequency is not absolute; it is contingent on volume, intensity, and recovery time [46].

Training frequency, as the only variable factor in a RT program, determines the total training volume When all other variables are fixed, frequency dictates how the total volume is distributed across the week and therefore governs the recovery interval between bouts [47].

Grgic et al. [48] reported that higher frequencies enhance strength in young adults, yet the same advantage was not observed in middle-aged and older sub-groups—probably because older adults recover more slowly from higher loads. Moreover, when total volume is held constant, the same work can be arranged in different ways (e.g., 4 × 5 vs. 2 × 10), and these configurations impose distinct acute stresses and, ultimately, different adaptations [47].

Currently, there is limited research on training frequencies exceeding three sessions per week for sarcopenic older adults. Typically, a training frequency of 2–3 times per week may be a critical point [49], with RT exceeding three sessions per week possibly having a negative impact on strength and functional improvement in sarcopenic older adults. Furthermore, it is challenging for older adults to adhere to a high-frequency RT program over the long term. Population-based studies demonstrate that engagement in RT is generally low among older adults [50], and some studies have even shown that RT once a week can improve muscle strength in older adults [51], although its effects are less than those seen with 2–3 sessions per week.

A large body of evidence shows that heavier loads are superior for maximizing strength because they elicit pronounced neural adaptations by requiring effort close to 1RM [52, 53].

In the present analysis, however, moderate-intensity training outperformed MVRT for improving grip strength. We attribute this finding to the choice of outcome: grip strength is rarely the primary target in high-load programmes, which typically focus on compound upper-limb movements. Consequently, high-load protocols may not allocate sufficient stimulus to the intrinsic hand and forearm musculature, whereas moderate-intensity routines—often designed with grip-intensive tasks—provide a more direct training stimulus, yielding larger gains in grip performance.

Chen et al. [21] reported that moderate-intensity training was more effective than MVRT in improving grip strength, which is consistent with the findings of this study. Currently, MVRT is more commonly applied in RT for the lower limbs in sarcopenic older adults, as improvements in lower limb strength are more closely related to gait, balance function, all-cause mortality, and Activities of Daily Living (ADL) scores [54, 55]. Some studies have reported [34] that no significant correlation was found between grip strength improvements and any other changes in physical fitness or overall function. Additionally, compared to upper body muscles, lower body muscles are larger and stronger, and they are more capable of adapting to high-intensity training, responding more positively to MVRT training intensity.

Among the 12 included trials, the participant pool is clearly skewed geographically: only two studies recruited Asian cohorts, while the remaining ten drew from Western populations. EWGSOP’s latest guidelines define muscle strength as the cardinal diagnostic criterion for sarcopenia [56]. prompting Western researchers to prioritise strength-focused interventions. Physiological differences reinforce this pattern: Caucasian sarcopenic adults typically present with both higher body mass and greater baseline grip strength than their Asian counterparts [57], factors known to amplify responsiveness to resistance training. Lifestyle divergences—including habitual physical-activity levels and dietary composition—may further modulate training outcomes. Consequently, our pooled results are likely to mirror the response profile of Western rather than Asian individuals, and caution is warranted when extrapolating these findings to other ethnic groups.

### Advantage

The principal strength of this review is its exclusive focus on the effects of pure resistance training in sarcopenic older adults, thereby isolating the impact of RT itself. Meta-regression further identified the strongest predictors of improvements in grip strength and SPPB scores, offering an evidence-based framework for optimising RT prescriptions.

### Limitations

Our study has several limitations that should be considered when interpreting the findings. First, despite a comprehensive search of international databases, our review was restricted to English and Chinese-language publications. This language bias may have omitted relevant studies published in other languages, potentially affecting the generalizability of our results. Second, the relatively small number of eligible trials not only reduces the statistical power of our analysis but also restricted our dose-response investigation to conventional variables (e.g., frequency, intensity, volume). Consequently, we were unable to explore the influence of other potentially important parameters, such as inter-set rest periods, time-under-tension, exercise tempo, and detailed exercise selection, which could provide more nuanced prescription guidelines. Third, although we performed a subgroup analysis on sarcopenic obesity, the small sample size limits the robustness of these findings and precludes any definitive conclusions for this specific population. A formal dose-response analysis was also not feasible. Finally, while meta-regression identified key moderators like frequency and intensity, this method cannot account for complex interactions between multiple training variables simultaneously. The overall low quality of evidence as assessed by the GRADE framework further necessitates a cautious interpretation of our results.

## Conclusion

In conclusion, the findings of this systematic review and meta-analysis suggest that pure RT is associated with improvements in grip strength and SPPB scores in older adults with sarcopenia, although its effect on SMI remains uncertain. Based on our dose-response findings, performing RT twice per week appears to be an effective strategy for enhancing grip strength, while a higher frequency (three times per week) did not yield additional benefits. Moderate intensity was identified as a sufficient training intensity for eliciting these improvements. Furthermore, incorporating pulling exercises into the RT regimen may positively influence grip strength outcomes. Despite these findings, the limitations of this study must be acknowledged, including the limited number of trials and the inability to control for all training parameters. Therefore, we cautiously suggest that clinicians and exercise professionals may consider prescribing moderate-intensity resistance training at a frequency of twice per week for older adults with sarcopenia, and that including pulling exercises could be beneficial. Future high-quality studies with larger sample sizes and more detailed exercise reporting are needed to confirm these associations and establish more precise prescription guidelines.

## Supplementary Information

Supplementary Material 1.

Supplementary Material 2.

## Data Availability

The datasets used and analysed during the current study are available from the corresponding author upon reasonable request.
