# Supplementary material for: Dose-response effects of resistance training in sarcopenic older adults: systematic review and meta-analysis
Source: BMC Geriatr. 2025 Nov 5;25:849. doi: 10.1186/s12877-025-06559-4 (PMC12590801; doi:10.1186/s12877-025-06559-4)
Supplement: Supplementary file 1 — Supplementary Material 1. [file 12877_2025_6559_MOESM1_ESM.pdf]

PubMed:

"exercise therapy"[All Fields] OR "exercising"[All Fields] OR "exercise s"[All Fields] OR "exercised"[All Fields] OR "exerciser"[All Fields] OR "exercisers"[All Fields]) OR ("exercise"[Mesh Terms] OR "exercise"[All Fields] OR ("physical"[All Fields] AND "activity"[All Fields]) OR "physical activity"[All Fields]) OR ("exercise"[Mesh Terms] OR "exercise"[All Fields] OR ("activities"[All Fields] AND "physical"[All Fields]) OR "activities physical"[All Fields]) OR ("exercise"[Mesh Terms] OR "exercise"[All Fields] OR ("activity"[All Fields] AND "physical"[All Fields]) OR "activity physical"[All Fields]) OR ("exercise"[Mesh Terms] OR "exercise"[All Fields] OR ("physical"[All Fields] AND "activities"[All Fields]) OR "physical activities"[All Fields]) OR ("exercise"[Mesh Terms] OR "exercise"[All Fields] OR ("exercise"[All Fields] AND "physical"[All Fields]) OR "exercise physical"[All Fields]) OR ("exercise"[Mesh Terms] OR "exercise"[All Fields] OR ("exercises"[All Fields] AND "physical"[All Fields]) OR "exercises physical"[All Fields]) OR ("exercise"[Mesh Terms] OR "exercise"[All Fields] OR ("physical"[All Fields] AND "exercise"[All Fields]) OR "physical exercise"[All Fields]) OR ("exercise"[Mesh Terms] OR "exercise"[All Fields] OR ("physical"[All Fields] AND "exercises"[All Fields]) OR "physical exercises"[All Fields]) OR ("exercise"[Mesh Terms] OR "exercise"[All Fields] OR ("acute"[All Fields] AND "exercise"[All Fields]) OR "acute exercise"[All Fields]) OR ("exercise"[Mesh Terms] OR "exercise"[All Fields] OR ("acute"[All Fields] AND "exercises"[All Fields]) OR "acute exercises"[All Fields]) OR ("exercise"[Mesh Terms] OR "exercise"[All Fields] OR ("exercise"[All Fields] AND "isometric"[All Fields]) OR "exercise isometric"[All Fields]) OR ("exercise"[Mesh Terms] OR "exercise"[All Fields] OR ("exercises"[All Fields] AND "isometric"[All Fields]) OR "exercises isometric"[All Fields]) OR

("exercise"[Mesh Terms] OR "exercise"[All Fields] OR ("exercise"[All Fields] AND "aerobic"[All Fields]) OR "exercise aerobic"[All Fields]) OR ("exercise"[Mesh Terms] OR "exercise"[All Fields] OR ("aerobic"[All Fields] AND "exercises"[All Fields]) OR "aerobic exercises"[All Fields]) OR ("exercise"[Mesh Terms] OR "exercise"[All Fields] OR ("exercise"[All Fields] AND "training"[All Fields]) OR "exercise training"[All Fields]) OR ("exercise"[Mesh Terms] OR "exercise"[All Fields] OR ("exercise"[All Fields] AND "trainings"[All Fields]) OR "exercise trainings"[All Fields])) AND ("sarcopenia"[Title/Abstract] OR "sarcopenias"[Title/Abstract]) AND ("randomized controlled trials as topic"[Mesh Terms] OR ("Controlled"[All Fields] AND "clinical trials as topic"[Mesh Terms]) OR "random*"[All Fields] OR "26"[All Fields] OR ("single blind method"[Mesh Terms] OR ("single blind"[All Fields] AND "method"[All Fields]) OR "single blind method"[All Fields] OR ("single"[All Fields] AND "blind"[All Fields] AND "method"[All Fields]) OR "single blind method"[All Fields]) OR ("double blind method"[Mesh Terms] OR ("double blind"[All Fields] AND "method"[All Fields]) OR "double blind method"[All Fields] OR ("double"[All Fields] AND "blind"[All Fields] AND "method"[All Fields]) OR "double blind method"[All Fields]))

Cochrane Library:

#1 Mesh descriptor: [Exercise] explode all trees 38838

#2 (Exercises or Physical Activity or Activities, Physical or Activity, Physical or Physical Activities or Exercise Physical or Exercises Physical or Physical Exercise or Physical Exercises or Acute Exercise or Acute Exercises or Exercise, Isometric or Exercises, Isometric or Exercise, Aerobic or Aerobic Exercises or Exercise Training or Exercise Trainings): ti,ab,kw (Word variations have been searched) 180407

#3 #1 OR #2 184187

#4 Mesh descriptor: [Sarcopenia] explode all trees 862

#5 (sarcopenias): ti,ab,kw (Word variations have been searched) 2334

#6 #4 OR #5 2334

#7 Mesh descriptor: [Randomized Controlled Trial] explode all trees 25729

#8 Mesh descriptor: [Controlled Clinical Trial] explode all trees 38474

#9 (random* or trial or Single-Blind Method or Double-Blind Method): ti,ab,kw (Word variations have been searched) 1476640

#10 #7 or #8 or #9 1476640

#11 #3 AND #6 1441

#12 #10 AND #11 1052

CNKI:

(resistance training OR physical exercise OR strength training OR aerobic exercise OR anaerobic exercise OR resistance exercise) AND （Sarcopenia OR sarcopenic obesity OR Geriatric Syndrome OR Muscle loss in the elderly OR Senile sarcopenia）

WANFANG:

(resistance training OR physical exercise OR strength training OR aerobic exercise OR anaerobic exercise OR resistance exercise) AND （Sarcopenia OR sarcopenic obesity OR Geriatric Syndrome OR Muscle loss in the elderly OR Senile sarcopenia）
